# Supplementary material for: Simple mapping-based quantification of a mock microbial community using total RNA-seq data
Source: PLoS One. 2021 Jul 16;16(7):e0254556. doi: 10.1371/journal.pone.0254556 (PMC8284643; doi:10.1371/journal.pone.0254556)
Supplement: Attachment — Submitted filename: ResponseToReviewer.docx [file pone.0254556.s004.docx]

Answer for Reviewer’s Comments to the Author

Reviewer #1:

The author reported a mapping-based approach for taxonomic annotation and quantification of RNA sequences. Here are my comments:

1. The method is of interest, but the manuscript in its present form is challenging to understand, making it impossible to assess. Thus, I would ask the author to engage a professional editor to improve the writing of the manuscript.

**Author response:** Thank you for your comments. I have hired a professional editor to improve the manuscript. In addition, abbreviations are re-organized, and I have added a table in the S1 file for this.

2. The author compared the performance of his method with Amplicon-Seq. Since multiple mapping-based taxonomic annotation and quantification methods already exist, the author should compare his method with the existing mapping-based methods.

**Author response:** I have added the MeTRS analysis, the only mapping-based method tested by mock and natural sample data. The MeTRS analysis pipeline is introduced, and the data has been analyzed. Results are described in the Results section and in Figs 2, 3, and 4. This method looks slightly better than MeTRS.

3. The cocktail of 10 bacterial cultures in no way reflects the real-world data. I would use a published dataset along with the dataset generated in the study.

**Author response:** The reported sequencing data was obtained using MeTRS from an original paper that used a human microbiome sample. The sequences were analyzed by the current study’s method and MeTRS, subsequently comparing them as described in the original data. Results are shown in the Results section and in the newly added Fig 5. Statistical analysis showed that both methods are significantly correlated.

4. BLAST is an acronym, hence replace blast+ with BLAST+.

**Author response**: I have replaced blast+ with BLAST+. Additionally, I have made changes in Figure 1, S1 Table, and the related main text to avoid confusion (abbreviation was changed from “blast” to “Bl”).

I suggest improving the writing of the manuscript with a professional editor and compare this new approach with the existing mapping-based approach using a robust real-world published dataset.

**Author response:** As mentioned above, I have made the following modifications, with appropriate descriptions.

1. A professional editor has edited the manuscript.
2. The current study’s method was compared with a well-validated RNAseq analysis method, MeTRS.
3. Both mock and “real-world” data were used for comparison between our method and MeTRS.
